# *Escherichia coli* O157:H7—Clinical aspects and novel treatment approaches

**DOI:** 10.3389/fcimb.2012.00138

**Published:** 2012-11-15

**Authors:** Elias A. Rahal, Natalie Kazzi, Farah J. Nassar, Ghassan M. Matar

**Affiliations:** Faculty of Medicine, Department of Experimental Pathology, Immunology and Microbiology, American University of BeirutBeirut, Lebanon

**Keywords:** *Escherichia coli* O157:H7, hemolytic uremic syndrome, hemorrhagic colitis, shiga toxins, antimicrobial chemotherapy

## Abstract

*Escherichia coli* O157:H7 is a notorious pathogen often contracted by intake of contaminated water or food. Infection with this agent is associated with a broad spectrum of illness ranging from mild diarrhea and hemorrhagic colitis to the potentially fatal hemolytic uremic syndrome (HUS). Treating *E. coli* O157:H7 infection with antimicrobial agents is associated with an increased risk of severe sequelae such as HUS. The difficulty in treating this bacterium using conventional modalities of antimicrobial agent administration has sparked an interest in investigating new therapeutic approaches to this bacterium. These approaches have included the use of probiotic agents and natural products with variable success rates. In addition, novel modalities and regimen of antimicrobial agent administration have been assessed in an attempt at decreasing their association with aggravating infection outcomes.

## General characteristics of *E. coli* O157:H7

The identification of *E. coli* O157:H7 as the etiologic agent of an outbreak of gastroenteritis that occurred in 1982 (Riley et al., 1983) has led to the recognition of a novel class of *E. coli*, the Enterohemorrhagic *E. coli* (EHEC). This group of pathogenic *E. coli* includes those that cause a clinical disease similar to that caused by *E. coli* O157:H7 and that possess few other characteristics of this organism, namely producing one or more phage-encoded Shiga toxins, possessing a hemolysin-encoding 60 MDa plasmid and that cause attaching and effacing (A/E)lesions (Levine, 1987; Nataro and Kaper, 1998).*E. coli* O157:H7 produces either or both of two toxins, one neutralized by antisera to shiga toxin produced by *Shigella dysenteriae* type 1 and referred to as Shiga toxin 1 (Stx1) while the other, Shiga toxin 2 (Stx2), is not neutralized by these antisera (Strockbine et al., 1986). Although *E. coli* O157:H7, like other *E. coli* ferments lactose, it does not ferment sorbitol within 48 h, unlike 80–95% of *E. coli* isolated from human stools (March and Ratnam, 1986). On the other hand, it does not grow well at 44–45.5°C, which is the default incubation temperature for detection of *E. coli* in food and water sources (Raghubeer and Matches, 1990).

Disease caused by *E. coli* O157:H7 has been reported from more than 30 countries on six continents (Doyle et al., 2001). In a 20-year surveillance period in the USA, 350 outbreaks were reported (Rangel et al., 2005). The Center for Disease Control and Prevention (CDC) estimates that *E. coli* O157: H7 causes 73,480 illnesses, 2168 hospitalizations and 61 deaths per year in the USA alone (Mead et al., 1999). *E. coli* O157:H7 has been found in cattle of several countries including the USA, Canada, Germany, Spain, England, and Scotland (Armstrong et al., 1996). Outbreaks have also occurred in these countries, as well as in Japan (Michino et al., 1999).

Cattle are considered to be the chief animal reservoir for *E. coli* O157:H7, which is a temporary member of their normal gut micro flora (Caprioli et al., 2005). *E. coli* O157:H7 has been isolated from many healthy cattle and has not been shown to be a pathogen in these animals. Cattle seem to lack vascular receptors for shiga-like toxins (Pruimboom-Brees et al., 2000). *E. coli* O157:H7 has also been isolated from other animals including deer (Diaz et al., 2011), sheep (Urdahl et al., 2003), horses (Lengacher et al., 2010), goats (Mersha et al., 2010), and dogs (Kataoka et al., 2010).

The first outbreak of *E. coli* O157:H7 occurred in 1982 and was traced to contaminated hamburger meat (Riley et al., 1983). Most outbreaks, particularly those that occurred during the 1980s were food borne with the main culprits being beef products particularly undercooked hamburgers in addition to unpasteurized milk (Griffin and Tauxe, 1991). During the past decade, however, marked changes in the epidemiology of human infections have taken place and outbreaks traced to vegetable and fruit sources, in addition to other food sources are on the rise. Infections traced to white radish sprouts (Michino et al., 1999), fresh spinach (Brandl, 2008), and lettuce (Hilborn et al., 1999). Consumption of tomatoes and apple juice has been frequently involved in outbreaks as well (McDowell and Sheridan, 2001). In addition, waterborne outbreaks have occurred (Swerdlow et al., 1992; Olsen et al., 2002; Bopp et al., 2003). *E. coli* O157:H7 appears to be capable of survival for prolonged times in water particularly at lower temperatures (Wang and Doyle, 1998). This microorganism was demonstrated to survive for more than eight months in a farm water gutter, and the surviving organisms were able to colonize cattle (Kudva et al., 1998). Swimming in contaminated water has also resulted in outbreaks (Keene et al., 1994; Friedman et al., 1999; Paunio et al., 1999). Person-to-person transmission has also been reported in day care centers and nursing homes as well (Panaro et al., 1990; Reida et al., 1994).

The rather easy spreading of *E. coli* O157:H7 from one person to another indicates that the infectious dose is rather low. Moreover, transmission by water, which would tend to dilute the organisms, substantiates this suggestion. The estimated infectious dose from outbreak data is 10–100 CFU (Griffin et al., 1994).

## Virulence factors of *E. coli* O157:H7

The ability to produce one or more shiga toxins is a hallmark *E. coli* O157:H7 infection. However, toxin production is not sufficient to cause disease. Two other factors are indicted in contributing to the virulence of *E. coli* O157:H7. The first of these two factors is harboring a 60 MDa virulence plasmid (pO157), which encodes a hemolysin (Schmidt et al., 1996; Mead and Griffin, 1998). The other factor is the locus of enterocyte effacement (LEE) (Kresse et al., 1998; Ogierman et al., 2000).

### The locus of enterocyte effacement (LEE)

The LEE contains all the genes necessary for inducing the A/E lesions typical of *E. coli* O157: H7 infection (Louie et al., 1993; Vallance and Finlay, 2000). As *E. coli* O157:H7 attaches to the gut mucosa and interacts with it, histopathological changes are produced in the epithelium. These changes are collectively known as A/E lesions (Kresse et al., 2000). These lesions are characterized by effacement of the epithelial brush border microvilli and the formation of actin-rich pedestals within the host cell underneath the attached bacterial cells. The presumed functions of these pedestals are prevention of dislodgement of the bacterium during the host diarrheal response and inhibition of bacterial phagocytosis (Devinney et al., 1999).

### pO157

All isolates of *E. coli* O157:H7 harbor the 60 MDa pO157 plasmid. This plasmid contains the *hly* operon encoding an enterohemolysin (Schmidt et al., 1996). This hemolysin, with the aid of specialized transport systems, may allow the bacterium to utilize the blood released into the intestine as a source of iron (Mead and Griffin, 1998).

### Shiga toxins

The Shiga toxin family comprises three members. Shiga toxin, produced by *Shigella dysenteriae* type 1, is the prototype Shiga toxin. On the other hand, Stx1 and Stx2 are produced by the EHEC. Several variants of Stx2 have been identified as well and these include Stx2c, Stx2d, Stx2e, Stx2f, and Stx2g. These share 84–99% of the amino-acid sequence of Stx2 but differ in some of its biological characteristics (Ito et al., 1990; Melton-Celsa and O'Brien, 1998; Schmidt et al., 2000; Melton-Celsa et al., 2002; Zheng et al., 2008). Three functional properties characterize the Shiga toxin family. These toxins are cytotoxic to HeLa and Vero cells. They lead to fluid accumulation in ligated rabbit illeal loops; therefore, they are “enterotoxic” and they are capable of inducing paralysis of the hind-legs and death in rabbit and mouse models (Jackson, 1990).

The binding moiety of these toxins, which aids them in binding to human and animal cells, consists of five B subunits. These subunits are non-covalently associated with an A subunit, which in turn consists of an A_1_ and an A_2_ subunit (Sandvig and Van Deurs, 1996). Shiga toxin and Stx1 differ only by a single amino acid in the B subunit (Calderwood et al., 1987; Hofmann, 1993). Thus, they are essentially identical; moreover, Stx1 is neutralized by antiserum to Shiga toxin (O'Brien and Holmes, 1987; Qadri and Kayali, 1998). Stx2 is antigenically distinct and unrelated. It is approximately 55% homologous to Shiga toxin/Stx1 (Jackson, 1990) and is not neutralized by antiserum to Shiga toxin (Qadri and Kayali, 1998).

The cellular receptors for the Shiga toxins are the neutral glycolipids globotriosylceramide (Gb3) and globotetraosylceramide (Gb4) (Betz et al., 2011). Various cell types are sensitive to Shiga toxins. These include enterocytes, renal, aortic, and brain endothelial cells, mesangial cells, renal tubular and lung epithelial cells, cells of the monocytic lineage, polymorphonuclear cells, in addition to platelets and erythrocytes among other cell types (Meyers and Kaplan, 2000).

After the toxin binds its receptor on the cell membrane, a short incubation leads to aggregation of toxin-receptor complexes in clathrin-coated pits. Next, the A fragment is endocytosed. The toxin is transported through endosomes to the Trans Golgi network (TGN). In the TGN, the toxin is cleaved by the enzyme furin into the A_1_ and A_2_ subunits. From the TGN, the toxin is transported to the endoplasmic reticulum where translocation into the cytosol takes place. If toxin was not cleaved by furin, then the cytosolic enzyme caplain may cleave the molecule (Hofmann, 1993; Sandvig and Van Deurs, 1996). The A_1_ subunit is a 28S rRNA N-glycosidase (Jackson, 1990). The toxin cleaves an adenine residue from a specific nucleotide of the 28S rRNA component of the 60S ribosomal subunit. This blocks tRNA binding to the 60S ribosomal subunit thus preventing peptide elongation and disrupting protein synthesis. This leads to cell death (Hofmann, 1993).

Shiga toxins induce an increase in chemokine synthesis from intestinal epithelial cells. This augments host mucosal inflammatory responses with release of interleukins, such as IL-8 and IL-1, in addition to Tumor Necrosis Factor (TNF). Activation of human endothelium by TNF or IL-1 leads to an increase in toxin receptor synthesis and hence increased sensitivity of the cell leading to increased cell death after exposure to the toxins (Meyers and Kaplan, 2000).

*E. coli* O157:H7 strains may produce either Stx1, Stx2, or both; however, most strains produce Stx2 (Mead and Griffin, 1998). Stx1 remains mostly cell-associated and stored in the periplasmic space while Stx2 is released from bacterial cells. Therefore, Stx1 is typically predominantly detected in cell lysates, while Stx2 is found in higher titers in culture supernatants (Strockbine et al., 1986; Yoh et al., 1997; Sato et al., 2003; Shimizu et al., 2009).

### Other virulence factors

While the LEE, pO157 and Shiga toxin production are defining virulence factors of *E. coli* O157:H7, other factors contribute to its pathogenicity. Some strains harbors EspP, which belongs to the family of serine protease autotransporters of Enterobacteriaceae (SPATE). This protease cleaves pepsin A and human coagulation factor V, which probably contributes to increased hemorrhage into the intestinal tract (Brunder et al., 1997). Moreover, EspP cleaves multiple complement system components hence protecting the bacterium from immune system-mediated elimination (Orth et al., 2010). On the other hand, in addition to LEE members such as intimin and Tir, bacterial attachment to host intestinal cells is also mediated by a type IV pilus referred to as the hemorrhagic coli pilus (HCP) (Xicohtencatl-Cortes et al., 2007). Multiple fimbriae and fimbrial gene clusters have also been implicated in contributing to adherence of this organism to host cells (Low et al., 2006).

## Clinical illnesses associated with *E. coli* O157:H7 infections

Infection with *E. coli* O157:H7 can be asymptomatic or may manifest as non-bloody diarrhea, hemorrhagic colitis, the hemolytic uremic syndrome (HUS), thrombocytopenia purpura and death (Griffin et al., 1988).

### Hemorrhagic colitis

Unless infection with *E. coli* O157:H7 is asymptomatic, following an incubation period of 3–4 days (Nauschuetz, 1998), the illness starts with severe abdominal cramps accompanied by a non-bloody diarrhea. In most patients the watery diarrhea becomes grossly bloody after two or three days (Boyce et al., 1995). Fever may be totally absent or may be of the low-grade type and its presence is more common in patients with severe illness (Griffin et al., 1988; Slutsker et al., 1997).

The duration of *E. coli* O157:H7 shedding seems to be age-dependent. Children under five years of age carry the organism after the resolution of symptoms longer than older children and adults (Pai et al., 1988). Intermittent shedding has also been reported (Belongia et al., 1993).

### The hemolytic uremic syndrome

Gastrointestinal symptoms due to infection with *E. coli* O157:H7 usually resolve within a week. Patients then mostly recover with no major sequelae. Nevertheless, 5–10% of patients under the age of 10 years develop the HUS approximately one week after onset of hemorrhagic colitis. The release of Shiga toxins is believed to play a central role in the development of HUS (Karmali et al., 1983). HUS most commonly occurs in children between 1 and 5 years of age but it can also occur in other groups particularly hospitalized patients over the age of 60 years. HUS displays a classical triad of microangiopathic hemolytic anemia (with fragmented RBCs on blood film), thrombocytopenia and renal failure (Gasser et al., 1955; Gianantonio et al., 1964). The patient's hematocrit may decline by 10%. Oligouria and hypertension (with elevated serum potassium, blood urea nitrogen, and uric acid levels) may occur as well. A condition known as thrombotic thrombocytopenia purpura (TTP) strikes mostly the adult population and is rarer than HUS. In TTP less marked renal damage is noted and fewer cases have a diarrheal prodrome. Both HUS and TTP can present with neurological abnormalities including seizures, coma and hemiparesis. These two conditions need not always be differentiated and may be referred to as HUS/TTP (Nauschuetz, 1998).

HUS and TTP are non-consumptive coagulopathies i.e., characterized by the consumption of platelets but not of clotting factors. They are regarded as variants of a single syndrome (Van Gorp et al., 1999). While fever and central nervous system (CNS) involvement are more frequent in TTP, renal dysfunction is less, and mortality and recurrences are greater. Although TTP can be initiated by *E. coli* O157:H7 infection, a diarrheal prodrome is uncommon (Siegler, 1995).

Classical postdiarrheal HUS always involves the colon and the kidney; however, other organ systems may be affected. The brain is most commonly affected with an evidence of CNS dysfunction in nearly one-third of HUS cases. Generalized seizures are common and occur in <20% of children affected. CNS injury symptoms range from disorders of posture, movement, and muscle tone to coma. Transient hepatocellular damage occurs in 40% of cases. The pancreas may be involved leading to diabetes mellitus, pancreatitis, and rarely, exocrine dysfunction. Other organs such as the heart, the lung and the skin are involved in rare cases (Siegler, 1995).

After the onset of the acute phase of HUS, characterized by the already mentioned triad of hemolytic anemia, thrombocytopenia, and acute renal injury, the patient's clinical disease may follow one of several patterns. More than 95% of cases recover from the acute phase of the disease. Thus, the mortality rate is 5% (McLigeyo, 1999). Grave sequelae, such as end-stage renal disease or permanent neurologic damage, occur in about 5% of subjects who survive the acute phase of HUS (Boyce et al., 1995).

Although an *E. coli* O157:H7 infection may result in HUS and TTP, numerous other causes may result in these diseases.

## Pathogenesis of *E. coli* O157:H7 infections

**Figure 1:**
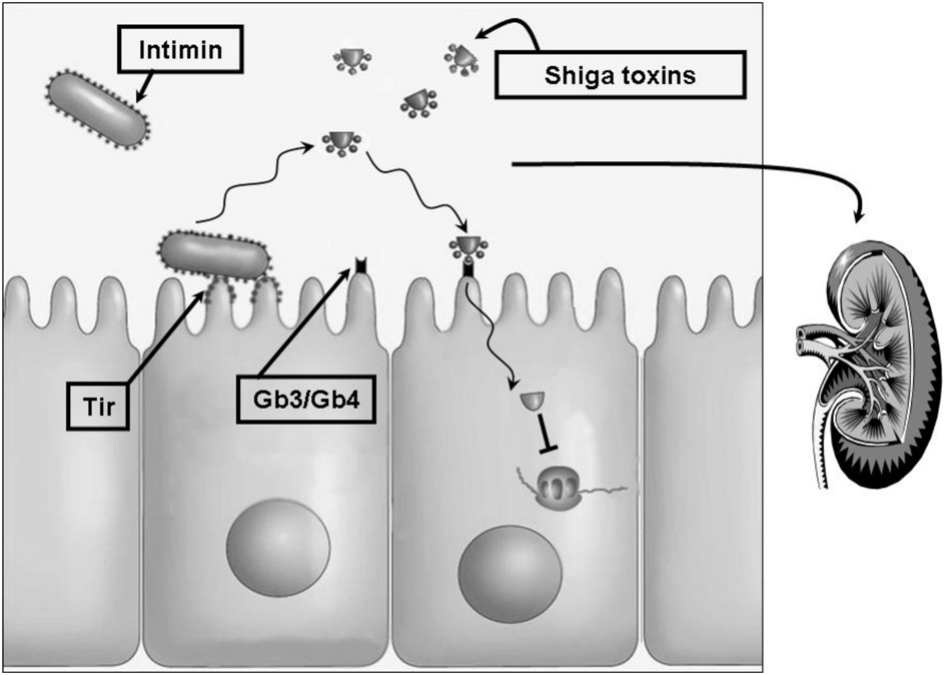
**The pathobiologic process of *E. coli* O157:H7.** The complex process by which *E. coli* O157:H7 attaches to the intestinal mucosa starts by bacterial fimbrial attachment followed by translocation of the bacterial Tir protein into the host cell membrane. Tir serves as the receptor for the bacterial outer membrane attachment protein intimin. One or more types of Shiga toxins are released which then bind to their cellular receptors, the neutral glycolipids Gb3 and Gb4. Internalization and cellular activation of these toxins blocks ribosomal peptide elongation hence disrupting protein synthesis leading to cell death. Intestinal damage permits Shiga toxins and other bacterial factors to gain entrance to the circulation. These may reach multiple host tissues including the kidneys where damage to the microvasculature results in the potentially lethal hemolytic uremic syndrome. Treatment of this disease remains largely supportive with no widely accepted antibacterial or toxin-targeted regimen. Antibacterial agents are believed to result in bacterial lysis and release of stored toxins. One potential treatment method may rely on inhibition of toxin expression prior to administration of a bactericidal agent.

The infectious process of *E. coli* O157:H7 (Figure 1) is initiated by the ingestion a relatively small inoculum of 10–100 CFUs. Only a few organisms are needed to allow enteric colonization (Mead and Griffin, 1998). The process by which these bacteria become attached to the mucosa of the distal ileum and the colon is complex and likely starts by bacterial fimbrial attachment followed by translocation of the bacterial Tir protein to the host cell membrane. Tir serves as the receptor for intimin, which is a bacterial outer membrane protein that plays a major role in attachment and production of A/E lesions characterized by effacement of microvilli (Devinney et al., 1999).

Owing to this bacterium's tremendous ability to produce the potent cytotoxic Shiga toxins, invasion of the host cells is not necessary for the progression to hemorrhagic colitis. Although the toxins are probably also not necessary for triggering the diarrhea, they most likely cause the intestinal lesions, characterized by hemorrhage and ulcerations, via damaging the microvasculature of the intestinal wall (Tesh and O'Brien, 1992).

Once the gut-blood barrier has been compromised by intestinal damage, Shiga toxins, and other bacterial products like lipopolysaccharide (LPS) gain entrance to the circulation. LPS can by itself, independent of Shiga toxins, damage endothelial cells, increase TNF levels, activate platelets and induce the blood coagulation cascade. It can also increase levels of interleukins such as IL-8, which is a potent activator of white blood cells (WBCs). WBCs participate in the pathogenic process by elaborating tissue-damaging enzymes such as elastase. Shiga toxins induce an increase in chemokine synthesis from intestinal epithelial cells probably augmenting host mucosal inflammatory responses with release of IL-8, TNF, and IL-1. As mentioned above, activation of human endothelium by TNF or IL-1 leads to an increase in expression of the Shiga toxin cellular receptors. This leads to an increased cell death after exposure to the toxins. Since the toxin receptors are widely distributed on various types of cells, thus many host tissues are affected (Meyers and Kaplan, 2000).

## Current and novel treatment approaches

Treatment of infection with EHEC strains, including *E. coli* O157:H7, is mainly based on supportive therapy, particularly rehydration. The use of antimotility agents, which inhibit peristalsis and delay clearance of the organism, poses a risk factor for progression to HUS (Cimolai et al., 1994; Bell et al., 1997). The use of antimicrobial agents in the treatment of *E. coli* O157:H7 infection is not recommended but remains a debatable issue (Safdar et al., 2002). This is based on studies that have shown it to be a risk factor for the development of HUS (Wong et al., 2000; Smith et al., 2012). Additionally, the use of trimethoprim, the quinolones, or furazolidone enhances the production of Shiga toxins from *E. coli* O157:*H7 in vitro* presumably due to lysis of bacterial cells and the release of stored toxins (Kimmitt et al., 2000). This enhanced release of toxins may alternatively be due to induction of Stx-producing prophages harbored by the bacterium. These prophages would be activated by the SOS response, a damage response triggered in these bacteria mostly due to genomic insult which may be exerted by antimicrobial treatment (Kimmitt et al., 2000).

In light of the difficulties in treating this agent, alternate treatment approaches were investigated by multiple groups. Antibodies to Stx2 were shown to enhance the survival of infected gnotobiotic piglets (Donohue-Rolfe et al., 1999). These antibodies were also demonstrated to be well tolerated in humans and thus may be useful for preventing HUS in pediatric subjects (Lopez et al., 2010). On the other hand, carbosilane dendrimers were shown to specifically bind to Shiga toxins with high affinity and to inhibit cellular entry of the toxin. Intravenous administration of these carbosilane dendrimers decreased the brain damage and prevented the lethal effect of the toxins in infected mice (Nishikawa et al., 2002).

The use of natural products for the treatment and prevention of *E. coli* O157:H7 has been assessed by multiple groups. Studies in infant rabbits show that the administration of *Lactobacillus casei*, commonly known for its probiotic efficiency, had a protective effect against the toxins of *E. coli* O157:H7 (Ogawa et al., 2001). Multiple other probiotic agents have been shown to be effective in curbing the growth or the pathogenic effect of this organism (Shu and Gill, 2001, 2002; Asahara et al., 2004; Takahashi et al., 2004; Gagnon et al., 2006; Kim et al., 2009; Etienne-Mesmin et al., 2011; Tahamtan et al., 2011). Certain herbs such as Chinese cinnamon, Spanish oregano and other essential oils have been shown to have mechanisms of action against the cell membrane and cell wall of *E. coli* O157:H7 (Oussalah et al., 2006). Green tea components (Lee et al., 2009) in addition to cranberry constituents (Lacombe et al., 2010) have also been shown to have an effect against this bacterium. In an attempt at implementing antimicrobial agents in the treatment of an *E. coli* O157:H7-infected animal model, azithromycin was shown to enhance the survival of infected piglets (Zhang et al., 2009). The majority of the studies mentioned herein have limited their testing to *in vitro* assays or employed animals that were gnotobiotic or treated with antimicrobial agents to limit the growth of their normal flora prior to infection with *E. coli* O157:H7. Consequently, the response to the tested agents in a host with a normal range of flora may be different.

Our group examined whether employing an agent that would inhibit toxin expression prior to treatment with a bactericidal antibiotic may be effective in treating such an infection. We assessed the effects of rifampicin, an RNA polymerase inhibitor, and gentamicin, a ribosome inhibitor, on the expression of the Stx1 and Stx2 encoding genes, *stx1* and *stx2* (Kanbar et al., 2003; Matar and Rahal, 2003; Rahal et al., 2011a,b). After incubation with antimicrobial agents, levels of *stx1* gene transcripts notably decreased by more than 99% in a sample treated with the minimum inhibitory concentration (MIC) of rifampicin, in that treated with the MIC of rifampicin followed by the minimum bactericidal concentration (MBC) of rifampicin and in the sample treated with the MIC of rifampicin followed by the MBC of gentamicin. The sample treated with the MBC of gentamicin alone showed a 51.37% decrease, which was the least noted toxin gene expression inhibition. A 77% decrease in *stx2* transcript detection was seen in the sample treated with the MBC of gentamcin alone. Samples treated with the MIC of rifmapicin, or with the MIC of rifampicin followed by the MBC of rifampicin, or with the MIC of rifampicin followed by the MBC of gentamicin showed complete inhibition of *stx2* transcript detection. Detection of toxin release from these bacteria using reverse passive latex agglutination (RPLA) yielded results that were mostly concurrent with the decrease in RNA synthesis except for samples treated with the MBC of gentamicin alone. Gentamicin seemed to trigger an enhanced release of toxins from treated cultures.

To assess the utility of an antimicrobial regimen for the treatment of an animal model of infection, multiple groups of BALB/c mice received 3× LD50 of an *E. coli* O157:H7 strain via intraperitoneal injection. These were then treated with various rifampicin/gentamicin regimen and were monitored for survival. None of the mice infected and left untreated and none of the mice infected but treated with the *in vivo* MBC equivalent dose of gentamicin survived. On the other hand, the highest survival rate was obtained with the group treated with the *in vivo* MIC equivalent dose of rifampicin followed by the *in vivo* MBC equivalent dose of gentamicin (50% survival rate). In comparison, 25% of the mice infected and treated with the *in vivo* MIC equivalent dose of rifampicin survived while mice treated post-infection with the *in vivo* MIC equivalent dose of rifampicin followed by the *in vivo* MBC equivalent dose of rifampicin showed a 12.5% survival rate.

Therefore, preliminary data support that antimicrobial agents may be used for the treatment of an *E. coli* O157:H7 infection. One promising treatment modality, as evidenced by our *in vivo* data, may be to implement an expression inhibitory dose of an agent that would limit toxin production prior to using a bactericidal dose of an antimicrobial. Such a treatment modality may also be of use in treating other Shiga toxin producing organisms including emerging agents such as *Escherichia coli* O104:H4; however, potential implementation of such a treatment remains to be assessed.

### Conflict of interest statement

The authors declare that the research was conducted in the absence of any commercial or financial relationships that could be construed as a potential conflict of interest.
